# Computationally efficient flux variability analysis

**DOI:** 10.1186/1471-2105-11-489

**Published:** 2010-09-29

**Authors:** Steinn Gudmundsson, Ines Thiele

**Affiliations:** 1 Center for Systems Biology, University of Iceland, Reykjavik, Iceland; 2 Faculty of Industrial Engineering, Mechanical & Industrial Engineering & Computer Science, University of Iceland, Reykjavik, Iceland

## Abstract

**Background:**

Flux variability analysis is often used to determine robustness of metabolic models in various simulation conditions. However, its use has been somehow limited by the long computation time compared to other constraint-based modeling methods.

**Results:**

We present an open source implementation of flux variability analysis called fastFVA. This efficient implementation makes large-scale flux variability analysis feasible and tractable allowing more complex biological questions regarding network flexibility and robustness to be addressed.

**Conclusions:**

Networks involving thousands of biochemical reactions can be analyzed within seconds, greatly expanding the utility of flux variability analysis in systems biology.

## Background

Flux balance analysis (FBA) [1,2] is concerned with the following linear program (LP)

(1) $$\begin{array}{ll} \max_{v} & c^{T}v\\ \text{subject to} & Sv\;=\;0\\ & v_{l}\leq \;v\;\leq \;v_{u} \end{array}$$

where the matrix **S **is an *m *× *n stoichiometry *matrix with *m *metabolites and *n *reactions and **c **is the vector representing the linear objective function. The decision variables **v **represent *fluxes*, with **V **⊆ ℝ*^n ^*and vectors **v***_l _*and **v***_u _*specify lower and upper bounds, respectively. The constraints **Sv **= **0 **together with the upper and lower bounds specify the *feasible region *of the problem.

Flux variability analysis (FVA) [3] is used to find the minimum and maximum flux for reactions in the network while maintaining some state of the network, e.g., supporting 90% of maximal possible biomass production rate.

Applications of FVA for molecular systems biology include, but are not limited to, the exploration of alternative optima of (1) [3], studying flux distributions under suboptimal growth [4], investigating network flexibility and network redundancy [5], optimization of process feed formulation for antibiotic production [6], and optimal strain design procedures as a pre-processing step [7,8].

Let **w **represent some biological objective such as biomass or ATP production. After solving (1) with **c **= **w**, FVA solves two optimization problems for each flux *v_i _*of interest

(2) $$\begin{array}{ll} {\text{max}}_{v}/\min_{v} & v_{i}\\ \text{subject to} & Sv\;\text{=}\;0\\ & w^{T}v\;\geq \;\gamma Z_{0}\\ & v_{l}\;\leq \;v\;\leq \;v_{u} \end{array}$$

where *Z*_0 _= **w***^T^***v**_0 _is an optimal solution to (1), *γ *is a parameter, which controls whether the analysis is done w.r.t. suboptimal network states (0 ≤ *γ <*1) or to the optimal state (*γ *= 1). Assuming that all *n *reactions are of interest, FVA requires the solution of 2*n *LPs. While FVA is clearly an embarrassingly parallel problem and is therefore ideally suited for computer clusters, this note focuses on how FVA can be run efficiently on a single CPU. A multi-CPU implementation of fastFVA can be done in the same way as for FVA, i.e., by distributing subsets of the *n *reactions to individual CPUs. It is expected to give almost linear speedup for sufficiently large problems.

## Implementation

A direct implementation of FVA iterates through all the *n *reactions and solves the two optimization problems in (2) from scratch each time by calling a specialized LP solver, such as GLPK [9] or CPLEX (IBM Inc.) At iteration *i*, *i *= 1, 2, ..., *n *all elements of **c **are zero except *c_i _*= 1. Since the only difference between each iteration is a change in the objective function, i.e., the feasible region does not change, solving the LPs from scratch is wasteful. Each time a LP is solved, the solver has to spend some effort in finding a feasible solution. Once a feasible solution is found, the solver then proceeds to locate the optimum. The small changes in the objective function suggest that, on average, the optimum for iteration *i *does not lie far away from the optimum for iteration *i *+ 1. With Simplex-type LP algorithms, this property can be exploited by solving problem (1) from scratch and then solving the subsequent 2*n *problems of (2) by starting from the last optimum solution each time (warm-starts). It should be noted that the default behavior of some Simplex-type solvers is to use warm-starts when a sequence of LPs is solved within the same application call. However, current implementations of FVA do not make use of this option (c.f. [10]). Furthermore, for increased efficiency, model preprocessing (presolving) should be disabled after solving the initial problem *P*. Given a value of 0 <*γ ≤ *1, fastFVA performs the following procedure

Setup problem (1), denote it by *P*

Solve *P *from scratch to get **v**_0 _and *Z*_0_

Add the constraint **w***^T^***v **≥ *γZ*_0 _to *P*

for *i *= 1 to *n*

   Let *c_i _*= 1 and *c_j _*= 0, ∀*j *≠ *i*

   Maximize *P*, starting from ***v****_i-_*_1_

                     to get **v***_i _*and *Z_i_*

   *maxFlux_i _*= *Z_i_*

Once all maximization problems have been solved, the minimization problems are solved in the same way, starting from **v**_0 _= **v***_n_*.

An important difference between the various LP solvers available is their ability to exploit multiple core CPUs or multi-processor CPUs to increase performance. The GLPK solver, for example, is a single threaded application. When running on a quad-core machine with hyperthreading enabled, the CPU load is only at 12-13%. On multi-core machines, a significant speedup can often be achieved by simply running multiple instances of fastFVA, each working on a different subset of the *n *reactions.

The fastFVA package runs within the Matlab environment, which will facilitate the use of fastFVA by users less experienced in programming. In addition, many biochemical network models can be imported into Matlab using the Systems Biology Markup Language (SBML) and the COBRA toolbox [10].

The fastFVA code is written in C++ and is compiled as a Matlab EXecutable (MEX) file (additional file 1). Matlab's PARFOR command is used to exploit multi-core machines. Two different solvers are supported, the open-source GLPK package [9], and the industrial strength CPLEX solver from IBM.

## Results and Discussion

We evaluated the performance of fastFVA on six biochemical network models ranging from approx. 650 up to 13,700 reactions (Table 1, additional file 2).

**Table 1:** The models used in the experiments

Model	References	Reactions	Metabolites
*T. maritima*	[13]	647	565
*P. putida*	[14]	1060	911
*E. coli*	[15]	2382	1668
Human	[16]	3820	2785
E-matrix	[17]	13694	11991
*E_coupled_*-matrix	[5]	13726	13047

### Performance evaluation

Four metabolic networks and two versions of a genome-scale network of the transcriptional and translational (tr/tr) machinery of *E. coli *were used for testing the fastFVA code (Table 1). The biomass reaction was used as an objective in the metabolic models, while the demand of ribosomal 50 S subunit was used as the objective in the tr/tr models. In all cases, flux distributions corresponding to at least 90% of optimal network functionality were sought.

The fastFVA code was tested on a DELL T1500 desktop computer with a 2.8 GHz quad core Intel i7 860 processor with hyperthreading enabled and Windows 7.

### Running times

The running times are given in Table 2 where fastFVA is compared to the direct implementation of FVA found in the COBRA toolbox [10]. The observed speedup is significant, ranging from 30 to 220 times faster for GLPK and from 20 to 120 times faster for CPLEX. The minimum and maximum flux values obtained with fastFVA were essentially identical to the values obtained with the direct approach (data not shown).

**Table 2:** Running time (s) for fastFVA versus a direct FVA implementation

	GLPK		CPLEX	
	FVA	fastFVA	FVA	fastFVA
*T. maritima*	10.3	0.3	4.3	0.2
*P. putida*	37.0	1.1	12.3	0.3
*E. coli*	340.0	2.5	119.5	1.5
Human	2217.8	12.5	659.8	5.4
E-matrix	12263.1	184.0	9514.6	108.1
*E_coupled_*-matrix	> 120 h	1919.4	30630.1	1421.7

### Other uses of fastFVA

The fastFVA code can be used to compute the flux-spectrum [11], a variant of metabolic flux analysis, simply by setting *γ *= 0 in (2). The *α*-spectrum [12], which has been used to study flux distributions in terms of extreme pathways, can also be computed with fastFVA. In this case, the parameter *γ *in (2) is set to zero and the **S **matrix is replaced by a matrix **P **containing the extreme pathways as its columns.

## Conclusions

With this efficient FVA tool in hand, new questions can be addressed to study the flexibility of biochemical reaction networks in different environmental and genetic conditions. It is now possible to design computational experiments requiring hundreds or even thousands of FVAs.

## Availability and requirements

The fastFVA package is freely available at http://notendur.hi.is/ithiele/software/fastfva.html together with pre-compiled binaries for Linux and Microsoft Windows. The fastFVA code runs under Matlab and relies on third-party solvers to solve linear optimization problems. Two such solvers are supported, the open source GLPK [9] and the industrial strength CPLEX (IBM Inc.) The fastFVA code is written in C++ and is compiled as a Matlab EXecutable function (MEX). It is released under GNU LGPL.

## Authors' contributions

IT conceived and designed the study. SG implemented fastFVA and carried out the experiments. Both authors wrote the manuscript and approved its final version.

## Supplementary Material

Additional file 1 **This file contains the C++ source code, the pre-compiled binaries, an example on how to use fastFVA and scripts for carrying out the experiments described above**. Click here for file

Additional file 2 **This file contains the six metabolic networks used in the experiments**. Click here for file
